# Seroprevalence of cytomegalovirus and rubella among pregnant women in western Sudan

**DOI:** 10.1186/1743-422X-8-217

**Published:** 2011-05-11

**Authors:** Hamdan Z Hamdan, Ismail E Abdelbagi, Nasser M Nasser, Ishag Adam

**Affiliations:** 1 Department of Biochemistry, Faculty of Medicine, Al-Neelain University, Khartoum, Sudan; 2 Faculty of Medicine, University of Khartoum, Khartoum, Sudan

## Abstract

**Background:**

Maternal cytomegalovirus (CMV) and rubella infections have adverse neonatal outcomes. Basic epidemiological data concerning CMV and rubella is necessary for health planners and care providers.

**Methods:**

A cross sectional study was conducted at El-Rahad hospital, Sudan to investigate seroprevalence of CMV and rubella infections and associated possible risk factors among pregnant women. Structured questionnaires were used to gather socio-demographic data and ELISA was used to detect CMV and rubella infections using IgG and IgM.

**Results:**

Out of 231 pregnant women, 167 (72.2%) and 151 (65.3%) were CMV-IgG and rubella-IgG positive, respectively. Only 6 (2.5%) and 8 women (3.4%) were CMV-IgM and rubella-IgM positive, respectively. While, high parity (OR = 14.7, 95%CI = 1.7 - 123.6; *P *= 0.01] and illiteracy (OR = 3.0, CI = 1.4 - 6.5; *P *= 0.004) were significantly associated with seropostive CMV-IgG in multivariate analysis, none of the other obstetrical and medical characteristics were significantly associated with CMV or rubella infections.

**Conclusion:**

CMV prevalence was 72.2% and rubella susceptibility among pregnant women was 34.6%. Rubella vaccine and routine screening for rubella and CMV should be introduced for pregnant women in this setting. Further research is needed.

## Introduction

Maternal Cytomegalovirus (CMV) is the commonest viral infection in perinatal period and it is the leading cause for congenital CMV infection with a permanent hearing, vision loss and neurological impairment [1-3]. It have been reported that, Africa continent have one of the highest prevalence of CMV e.g. in neighboring Egypt, CMV seroprevalence among pregnant women was 96% [3,4]. Maternal sexual behavior and contact with infected young children were the known source of infection [5]. While CMV has asymptomatic infection, rubella infection is mild or self limiting disease, transmitted through respiratory system and to growing fetus through placenta [6,7]. Maternal infection especially during the first trimester associated with adverse neonatal outcome which encompass heart disease, cataract and deafness collectively known as congenital rubella syndrome which had a major neonatal morbidity and burden to families [8]. Although, incidence of rubella infection is reduced worldwide, some African countries like Mozambique still has a high incidence (95.3%) [9,10]. Rubella vaccine is cost-effective and cost-beneficial, therefore since year 2000 WHO proposed an introduction of rubella vaccine program in each country [11].

There is no published data concerning CMV and rubella seroprevalence in pregnant women in Sudan. The basic data concerning CMV and rubella infections during pregnancy is important for health planners and care providers. Thus, this was the aim of the current study as to investigate seroprevalence, associated possible risk factors for CMV and rubella infections among pregnant women in west Sudan. This work is the part of collaborative projects between University of Khartoum and Ministry of Health Sudan so as to provide the later with basic data necessary for intervention [12].

## Methods

This was a cross-sectional study conducted at Antenatal Care Clinic of El-Rahad hospital, western Sudan during the period of August - October 2009. Consecutive pregnant women were approached to participate in the study. After signing an informed consent, relevant medical, obstetrical, socio-demographic characteristics were gathered using pre-tested questionnaires. Women were inquired for history of Jaundice and miscarriage. Body mass index (BMI) was calculated by measuring weight and height. Five mls of blood were collected in plain tubes, allowed to clot and centrifugated at room temperature. Then sera were stored at -20°C till transported to Khartoum in dry ice for analyses. Enzyme-linked immunosorbent assay (ELISA) was used for CMV and rubella (IgG and IgM) using commercial diagnostic kits (DRG Instruments GmbH. Germany). Quantitative analysis for CMV and rubella (IgG and IgM) were performed, and the assay result interpreted as IU/mL. The manufacturer's instructions were followed for the cutoff points, which was < 9 IU/mL for CMV IgG and IgM. Results < 10 and (< 68 IU/mL was considered negative for rubella IgG and IGM, respectively.

### Statistics

Data were entered in the computer using SPSS for windows version16.0 and double checked before analysis. Means and proportions of the socio-demographic and obstetrical characteristics were calculated for CMV and rubella seropostive groups. Univariate and multivariate analysis were used for CMV and rubella IgG seropostive groups as dependent variable and socio-demographic and obstetrics variables as independent variables. *P *value < 0.05 was considered significant.

## Result

### Socio-demographic and clinical characteristics

A total of 231 pregnant women were enrolled to the study. The mean (SD) of age, parity and gestational age were 25.7 (5.9) years, 2.5 (2.1), 25.5(9.6) weeks, respectively. More than third (39.4%) of these women were illiterate. Forty-five (19.5%) women has history of miscarriage and 60 (26%) had past history of jaundice, table 1.

**Table 1:** Obstetrical, socio-demographical and clinical characteristic of the pregnant women in El-Rahad Hospital Western Sudan

Variables	Total (*N*= 231)	Cytomegalovirus seropostive (*N*= 167)	Rubella seropostive (*N *= 151)
Age (years)	25.7 (5.9)	26.5 (5.94)	25.6 (5.8)
Haemoglobin (g/dl)	11.1 (1.6)	11.09 (1.6)	11.1 (1.6)
Parity	2.5 (2.1)	3.09 (2.1)	2.5 (2.1)
Gestational age (weeks)	25.5 (9.6)	26.13 (9.4)	26.24 (9.3)
Body mass index	24 (23)	24.65	23 (4.8)
Education level			
Illiterate	91 (39.4)	78 (46.7)	65 (43)
Primary	100 (43.3)	70 (41.9)	67 (44.4)
Secondary	31 (13.4)	17 (10.2)	14 (9.3)
University	9 (3.9)	2 (1.2)	5 (3.3)
Occupation			
House wife	104 (45.2)	72 (43.1)	69 (45.7)
Farmer	114 (49.4)	90 (53.9)	76 (50.3)
Employee	12 (5.2)	5 (3)	6 (4)
History of Miscarriage			
Yes	45 (19.5)	38 (22.8)	31 (20.5)
No	179 (77.5)	126 (75.4)	117 (77.5)
History of Jaundice			
Yes	60 (26)	42 (25.1)	40 (26.5)
No	171 (74)	125 (74.9)	111 (73.5)

Data are expressed as Mean (SD) or number (percentage).

### CMV and rubella seroprevalence

Out of these 231 pregnant women, 167 (72.2%) and 6 (2.5%) had seropostive CMV IgG and IgM, respectively. A total 151 (65.3%) and 8 (3.4%) women had seropostive rubella IgG and IgM, respectively. For both CMV and rubella, those women with IgM positive had IgG positive too. One hundred-eight (46.75%) women had IgG positive for both CMV and rubella.

### Risk factors for CMV and rubella virus

Univariate and multivariate analysis showed that both illiteracy (OR = 3.0, CI = 1.4-6.5; *P *= 0.004) high parity [> 5 deliveries] (OR = 14.7, CI = 1.7 - 123.6; *P *= 0.01) were significant risk factors for CMV infection. Age was significantly associated with CMV infection in univariate analyses. Gestational age, history of miscarriage, past history of jaundice, hemoglobin level and body mass index were not significantly associated with CMV infection, table 2. While illiteracy was significantly associated with rubella infection in univariate analyses, none of the investigated socio-demographic obstetrical factors were associated with rubella in multivariate analyses, table 3.

**Table 2:** Factors associated with cytomegalovirus (CMV) infection in pregnancy in El-Rahad hospital, Western Sudan, using Univariate and multivariate analysis.

Variables	Univariate analysis			Multivariate analysis		
	
	OR	95% CI	*P value*	OR	95% CI	*P value*
Age	1.1	1.0 - 1.1	<0.001	1.0	0.9 - 1.0	0.6
Illiteracy	3.4	1.7 - 6.7	<0.001	3.0	1.4 - 6.5	0.004
Gestational age	1.0	0.9 - 1.0	0.1	1.0	0.9 - 1.0	0.3
High parity	22.5	3.0 - 167.3	0.002	14.7	1.7 - 123.6	0.01
History of miscarriage	2.2	0.9 - 5.4	0.06	1.5	0.5 - 4.4	0.3
History of jaundice	0.8	0.4 - 1.6	0.6	0.5	0.2 - 1.1	0.1
Haemoglobin	0.9	0.7 - 1.0	0.2	0.8	0.6 - 1.0	0.2
Body mass index	1.0	0.9 - 1.0	0.6	1.0	0.9 - 1.0	0.4

Abbreviations: OR, Odds Ratio; CI, confidence interval

**Table 3:** Factors associated with Rubella virus infection in pregnancy in El-Rahad hospital Western Sudan, using Univariate and multivariate analysis.

Variables	Univariate analysis			Multivariate analysis		
	
	OR	95% CI	*P value*	OR	95% CI	*P value*
Age	0.9	0.9 - 1.0	0.7	1.0	0.9 - 1.1	0.4
Educational level	0.6	0.4 - 0.9	0.02	0.6	0.4 - 1.0	0.08
Gestational age	1.0	0.9 - 1.0	0.1	1.0	0.9 - 1.0	0.4
Parity	1.0	0.8 - 1.1	0.9	0.9	0.7 - 1.1	0.5
History of miscarriage	1.1	0.5 - 2.3	0.6	1.2	0.5 - 2.8	0.5
History of jaundice	1.0	0.5 - 2.0	0.8	1.2	0.6 - 2.5	0.4
Haemoglobin	0.9	0.7 - 1.1	0.4	0.9	0.7 - 1.0	0.2
Body mass index	0.9	0.9 - 1.0	0.3	0.9	0.9 - 1.0	0.5

Abbreviations: OR, Odds Ratio; CI, confidence interval

## Discussion

To our knowledge this the first published data in Sudan concerning epidemiology of CMV and rubella among pregnant women. In the current study, the prevalence of CMV IgG was 72.2%. The prevalence of CMV IgG among pregnant women was reported to be higher in other African countries e.g. 97.2% in Benin [13], 96% in Egypt [4], and 87% in Gambia [14]. However, much higher prevalence of CMV was reported in South East Asia [15], while European countries show low prevalence [16]. The low prevalence of CMV in this setting could be explained; firstly by the difference of HIV (which is important co-infection with CMV) prevalence in these settings [17-21]. We have recently observed a low HIV prevalence among pregnant Sudanese women [18]. Secondly, the different socio-demographic, various cultures and behaviors among these settings might have influence and determine epidemiology of CMV e.g. practice of breast feeding, child care and sexual activity [22-24].

Rubella prevalence in this study was 65.3%, hence 34.7% of the pregnant women are at risk for rubella infection and their unborn babies are vulnerable to congenital rubella syndrome. However, this prevalence is in concert with those reported from Nigeria 68.5% [25]. Although, there is a high prevalence of CMV all over the world, there is no available vaccine for CMV up to the moment. On the other hand, rubella vaccine - is not yet recommended in Sudan-has been licensed since 1969 [26].

In the current study illiterate women and women with high parity were at higher risk for CMV infection. High parity and illiteracy were observed before as risk factors for increased susceptibility to acquisition CMV infection, perhaps through the direct contact with contagious secretions from their own children and poor hygiene practiced by these women [27-30]. Likewise, low socio-economic status has been found as a strong risk factor for acquisition CMV infection [28]. Nevertheless in Sudan it is difficult to investigate the socio-economic status of these pregnant women because the culture is based on generous hospitality attitude toward guest and family members who usually lives in extended families.

In the current study age was not significantly associated with CMV or rubella infections. There is a lot of debate concerning maternal age and CMV infection; while many investigators observed that, elder women were at higher risk of CMV infection [28], others reported the reverse [31]. However, Bukbuk et al., documented that, increasing maternal age was significantly associated with rubella infection among Nigerian women [32].

The current study has many limitations; one of these we did not use Polymerase Chain Reaction (PCR) of the viral DNA isolation. The other limitation is the lack of follow-up for these women in order to document seroconveration and to detect fetal infections.

## Conclusion

This study show the prevalence of 72.2% and 65.3% of CMV and rubella infections among pregnant women in western of Sudan respectively, illiteracy and high parity are the risk factors for CMV infection. Rubella vaccine is recommended for childbearing age women. More research is needed.

## Competing interests

The authors declare that they have no competing interests.

## Ethics

This study was approved by Sudan Medical specialization Ethics Review Board, Sudan.

## Authors' contributions

HZH and IEA carried out the study and participated in the statistical analysis and procedures. NMM carried out the practical part of the study. IA coordinated and participated in the design of the study, statistical analysis and the drafting of the manuscript. All the authors read and approved the final version.
